# Allogeneic Human Bone Marrow‐Derived Mesenchymal Stem Cells Accelerate Diabetic Wound Healing via Inflammation Suppression and Angiogenesis Promotion: A Preclinical Study

**DOI:** 10.1002/kjm2.70303

**Published:** 2026-10-15

**Authors:** Rong‐Fu Chen, Keng‐Fan Liu, Chia‐Chun Lee, Yu‐Han Chen, Hsin‐Hsin Shen, Yur‐Ren Kuo

**Affiliations:** ^1^ Division of Plastic Surgery, Department of Surgery Kaohsiung Medical University Hospital Kaohsiung Taiwan; ^2^ Faculty of Medicine, College of Medicine, Orthopedic Research Center, Regenerative Medicine and Cell Therapy Research Center Kaohsiung Medical University Kaohsiung Taiwan; ^3^ Biomedical Technology and Device Research Laboratories Industrial Technology Research Institute Hsinchu Taiwan; ^4^ Department of Biological Sciences National Sun Yat‐Sen University Kaohsiung Taiwan; ^5^ Academic Clinical Programme for Musculoskeletal Sciences Duke‐NUS Graduate Medical School Singapore Singapore

**Keywords:** allogeneic cell therapy, diabetic wound healing, human mesenchymal stem cells, inflammation modulation, regenerative medicine

## Abstract

Mesenchymal stem cells (MSCs) have been shown to enhance tissue regeneration and wound healing. This preclinical study investigated the therapeutic potential of human bone marrow‐derived mesenchymal stem cells (hBM‐MSCs) for accelerating diabetic wound healing. Isolated hBM‐MSCs were characterized for surface marker expression and differentiation potential. Their migration‐promoting effects were evaluated in vitro. For the in vivo study, a dorsal skin defect model was created in streptozotocin (STZ)‐induced diabetic rats. Rats received periwound subcutaneous injections of low‐ or high‐dose hBM‐MSCs on Days 4 and 7, or freezing fluid as a vehicle control. Wound healing progression was assessed clinically, and tissue biopsies were analyzed using immunohistochemistry to evaluate inflammatory cell infiltration, cellular proliferation, and angiogenesis. hBM‐MSCs exhibited the expected surface marker profile and multilineage differentiation potential. In vitro, hBM‐MSCs significantly enhanced the migration of human umbilical vein endothelial cells and human foreskin fibroblasts. In vivo, wounds treated with hBM‐MSCs showed significantly accelerated healing compared to vehicle‐treated controls (*p* < 0.05). Immunohistochemical analysis revealed reduced inflammatory cell infiltration and increased expression of Ki‐67, epidermal growth factor (EGF), vascular endothelial growth factor (VEGF), and prolyl 4‐hydroxylase (P4H) in hBM‐MSC‐treated wounds. Allogeneic hBM‐MSCs significantly promote diabetic wound healing by suppressing inflammation, enhancing cellular proliferation and differentiation, and stimulating angiogenesis. These findings highlight the therapeutic potential of allogeneic hBM‐MSCs for treating diabetic wounds, particularly in patients unsuitable for autologous cell therapy.

## 1 Introduction

Diabetes mellitus (DM) presents a burgeoning concern due to the considerable threat it poses to both mortality and morbidity, chiefly manifested through the occurrence of diabetic foot ulcers (DFUs) and related complications [1]. Refractory diabetes wounds are an unmet therapeutic area since their assessment and management are extremely complex and stand as a primary instigator for complications such as infection and necessitated amputations, consequently imparting a substantial burden on healthcare systems and associated psychosocial and economic costs [2, 3].

Unhealed chronic wounds exhibit common features typified by heightened levels of proinflammatory cytokines, proteases, and reactive oxygen species (ROS), which cause insufficient blood perfusion to the wound bed and decrease cell proliferation and migration, which are the key underlying factors in poor diabetic wound healing [4]. It is crucial to seek effective interventions to treat such debilitating wounds.

Studies showed that mesenchymal stem cells (MSCs) have garnered considerable attention for their regenerative properties in wound healing. Numerous strategies have been discussed, but in some cases, contriversing results still showed. In vivo studies have demonstrated the efficacy of exogenous MSCs in promoting wound healing and mitigating scarring, owing to their broad differentiation potential and regenerative capabilities. Consequently, MSCs have emerged as a promising therapeutic avenue in the realm of regenerative medicine, particularly in the treatment of chronic wounds [5, 6, 7]. Previous research has showcased the potential of adipose‐derived stromal cells (ADSCs) and bone marrow‐derived mesenchymal stem cells (BM‐MSCs) in enhancing diabetic wound healing in rodent models [8, 9]. Given the time‐intensive nature of autologous MSC expansion, this study aimed to evaluate the therapeutic efficacy of human allogenic BM‐MSCs (hBM‐MSCs) in vitro and investigated the effects of treating diabetic wounds in a rodent wounding model for a preclinical study.

## 2 Methods

### 2.1 Isolation and Cell Culture of hBM‐MSCs

The frozen hBM‐MSCs (provided by the Industrial Technology Research Institute), comprising 1.5 × 10^7^ cells in 20 mL of normal saline, were carefully thawed and centrifuged to remove the cell storage solution. Cells were then expanded in a culture medium containing PRIME‐XV MSC Expansion XSFM (FUJIFILM Irvine Scientific) and maintained at 37°C in a humidified 5% carbon dioxide atmosphere [10]. The subcultures were performed with TrypLE Select (Gibco, USA) before reaching 100% confluence.

### 2.2 Characterization and Differentiation Assays of hBM‐MSCs

The cultured hBM‐MSCs were treated with an induced medium to investigate the adipogenic, osteogenic, and chondrogenic differentiation potential based on the experimental methods published by Pittenger et al. [11] and Lin et al. [12]. Trypsinized hBM‐MSCs were washed and separated into an appropriate cell concentration in magnetic activated cell sorting (MACS) separation buffer (Miltenyi Biotec, Germany). The identification of hBM‐MSCs surface markers was performed by adding fluorescent conjugated anti‐human CD73, CD90, CD105, HLA‐ABC, CD34, CD45, CD19, CD14, and HLA‐DR antibodies (BD Pharmingen, USA; exclusive of anti‐human CD105 antibody, which was from Bio‐Rad, USA) [13, 14]. After incubation at 4°C for 30 min with light protection, staining samples were washed with MACS separation buffer and resuspended in Dulbecco's phosphate‐buffered saline (DPBS; Gibco, USA) for the following analysis. The fluorescence intensity of each surface marker was measured in BD Accuri C6 flow cytometer (BD Biosciences, USA). The analyzed records were illustrated as histograms using BD Accuri C6 Software (BD Biosciences, USA).

### 2.3 hBM‐MSCs Conditioned Medium (hBM‐MSCs‐CM)

hBM‐MSCs were seeded at the density of 2.5 × 10^4^ cells/cm^2^ in 12‐well CellBIND Clear Multiple Well Plates (Corning, USA). After harvesting for 24 ± 2 h until cell adherence, the culture medium was discarded, followed by 1 mL DPBS washing. The fresh culture medium, medium 199 (M‐199; Gibco, USA), was added into each well to a final volume of 1 mL. The conditioned medium was obtained 24 ± 2 h later, separately, and stored at −80°C.

### 2.4 HUVECs and HFFs Migration Assay

Human umbilical vein endothelial cells (HUVECs) and human foreskin fibroblast (HFFs) were purchased from the Taiwan Medical Cell and Bioresource Collection and Research Center (BCRC, Taiwan). Cells were maintained and passaged at 37°C in a humidified 5% carbon dioxide atmosphere by medium 199 (M‐199; Gibco, USA) supplemented with 10% fetal bovine serum (FBS; Gibco, USA). The migration assay was designed for three experimental groups according to the difference in content of the lower chamber: Serum‐free M‐199 (control), 10% FBS‐supplemented M‐199 (positive control), and hBM‐MSCs‐CM. The medium of each condition (450 μL) was added into the lower chamber, each well of the 24‐well plate. HUVECs (2.5 × 10^4^ cells) resuspended in culture medium (250 μL) were initially transferred into the upper chamber devices (Transwell Permeable Supports, Corning, USA). After 16–18 h of incubation, HUVECs migrated to the bottom surface of the transwell membranes and were fixed with 4% Paraformaldehyde (PFA; ChemCruz Biochemicals, Netherlands) for 1 h at room temperature, followed by cell staining with 1% crystal violet dye. The remaining HUVECs in the upper chamber were scraped out by the swabs, and stained cells were captured by Nikon ECLIPSE Ti‐S Inverted Microscopes afterward. The average number of migrated cells was calculated in five random fields through the application of 4′,6‐diamidino‐2‐phenylindole (DAPI, Sigma‐Aldrich, St. Louis, MO) staining, Zeiss Axiovert 200 M Fluorescence Microscope, and Image J software.

### 2.5 HFFs Transwell Migration Assay

Two hundred microliters of collagen solutions (8.25 μg/mL) prepared with Collagen type I rat tail (Corning) and PBS were placed into the transwell device (Transwell Permeable Supports, Corning, USA), and the solidification was performed at 37°C for 1 h. 2.5 × 10^4^ cells of HFFs were then seeded in the transwell device in 250 μL of culture medium. Similarly, there were three different groups, including serum‐free M‐199 (control), 10% FBS‐supplemented M‐199 (positive control), and hBM‐MSCs‐CM as the testing sample. The following methods were described in the HUVECs migration assay above.

### 2.6 Animal Model and Experimental Procedures

All animal procedures adhered to the guidelines outlined in the National Institutes of Health's Guide for the Care and Use of Laboratory Animals. Animals were bred under standard conditions at the Center for Laboratory Animals, Kaohsiung Medical University, and this study was approved by the Institutional Animal Care and Use Committee (IACUC) of Kaohsiung Medical University Chung‐Ho Memorial Hospital (KMUH).

Type I DM was induced in 2‐month‐old male Wistar rats (BioLASCO Taiwan Co. Ltd., Taipei, Taiwan) via a single intraperitoneal injection of streptozotocin (STZ; 65 mg/kg; Sigma‐Aldrich, St. Louis, MO, USA) [15, 16]. Hyperglycemia was confirmed by plasma glucose levels exceeding 300 mg/dL 1 week post‐STZ injection. Diabetic rats were monitored for at least 4 weeks to assess the development of diabetic complications before surgery. To maintain stable blood glucose levels within the 200–300 mg/dL range, animals received intermittent insulin injections (1–2 units/kg; Novo Nordisk A/S) based on blood glucose monitoring.

A full‐thickness skin defect (6 × 5 cm^2^) was created on the dorsal skin of diabetic rats following standard surgical procedures as described in our former research [8, 17]. The wound bed was sutured with 4–0 silk sutures and covered with a transparent Tegaderm (3 M HealthCare, Borken, Germany) dressing. Postoperatively, rats were housed individually and antibiotics (ampicillin, 50 mg/kg/day; Y F Chemical Corp., New Taipei, Taiwan) were administered for 3 days. Wound healing progress was monitored weekly by measuring the wound area.

A total of 28 male Wistar rats were randomly assigned to four experimental groups (Each subgroup *n* = 7). General anesthesia was induced using isoflurane (Forane, AbbVie, Austria) for all animals.

*Group I (Sham control):* Diabetic rats underwent wound creation but received no cell treatment.

*Group II (diabetic control):* Diabetic rats underwent wound creation and subcutaneous injection of freezing fluid.

*Group III (hMSC‐L, low‐dose):* Diabetic rats underwent wound creation and received two subcutaneous injections of 1.4 × 10^6^ hBM‐MSCs in 1.6 mL normal saline at the wound margin on postoperative Days 4 and 7.

*Group IV (hMSC‐H, high‐dose):* Diabetic rats underwent wound creation and received two subcutaneous injections of 7.7 × 10^6^ hBM‐MSCs in 1.6 mL normal saline at the wound margin on postoperative Days 4 and 7.

### 2.7 Histological and Immunohistochemical Analysis

To assess wound tissue architecture and cellular changes during healing, full‐thickness (3 mm) biopsies were collected from the wound margins at 1.5 and 2.5 weeks posttreatment. Tissue samples were fixed in 10% formalin (Sigma‐Aldrich, St. Louis, MO, USA) and embedded in paraffin for subsequent histological analysis. Sections for each experimental group underwent staining with hematoxylin and eosin (H&E; Sigma‐Aldrich). Immunohistochemistry (IHC) semiquantitative staining was carried out using the horseradish peroxidase–diaminobenzidine (HRP–DAB) staining kit (R&D Inc., Minneapolis, MN, USA), following established protocols [8, 18]. Polyclonal antibodies targeting CD45, vascular endothelial growth factor (VEGF), Ki‐67, epidermal growth factor (EGF), and prolyl 4‐hydroxylase (P4H) (Santa Cruz, Santa Cruz, CA, USA) served as primary antibodies, and the sections were incubated with these antibodies at a 1:100 dilution in phosphate‐buffered saline for 1 h. Subsequently, the sections underwent incubation with biotinylated goat anti‐rabbit antibodies for 30 min. The specific binding of secondary antibodies to primary antibodies was visualized using HRP, facilitating the enzymatic conversion of the chromogenic substrate 3,3′‐diaminobenzidine (DAB) into a brown precipitate. Following mounting, clearing, and coverslipping, the sections were examined using a Zeiss fluorescence microscope (Carl Zeiss, Gottingen, Germany).

Tissue sections were captured and analyzed using a Zeiss Axioskop 2 plus microscope (Carl Zeiss) and a Cool CCD camera (SNAP‐Pro cf. Digital kit; Media Cybernetics, Silver Spring, MD, USA). Image‐Pro Plus image analysis software (Media Cybernetics) was employed for image analysis, as previously described. Four random images from each selected area were acquired at ×400 magnification, and the quantification of immunopositive cells, along with the percentage of positively labeled cells to the total cells, was presented for further interpretation.

### 2.8 Statistical Analysis

The data were collected and then recorded on a spreadsheet (Microsoft Excel, Redmond, WA, USA). Statistical analysis was performed by using SPSS (SPSS Inc., Chicago, IL, USA). Response percentages were calculated, and the Chi‐square test was applied to compare responses between each category. *p* < 0.05 was considered statistically significant.

## 3 Results

### 3.1 Characterization of hBM‐MSCs

Human BM‐MSCs were isolated, cultured in vitro, and characterized based on surface markers and multi‐lineage differentiation potential. Flow cytometry analysis revealed high expression levels of CD90 (99.9%), CD73 (99.8%), CD105 (97.66%), and HLA‐ABC (98.78%), minimal expression of CD45 (0.04%) and CD34 (0.08%), and no expression of CD14, CD19, and HLA‐DR (Figure 1A). When subjected to conditions promoting mesenchymal lineage differentiation, hBM‐MSCs demonstrated osteogenic, adipogenic, and chondrogenic lineage phenotypes compared to the control group (Figure 1B).

**FIGURE 1:**
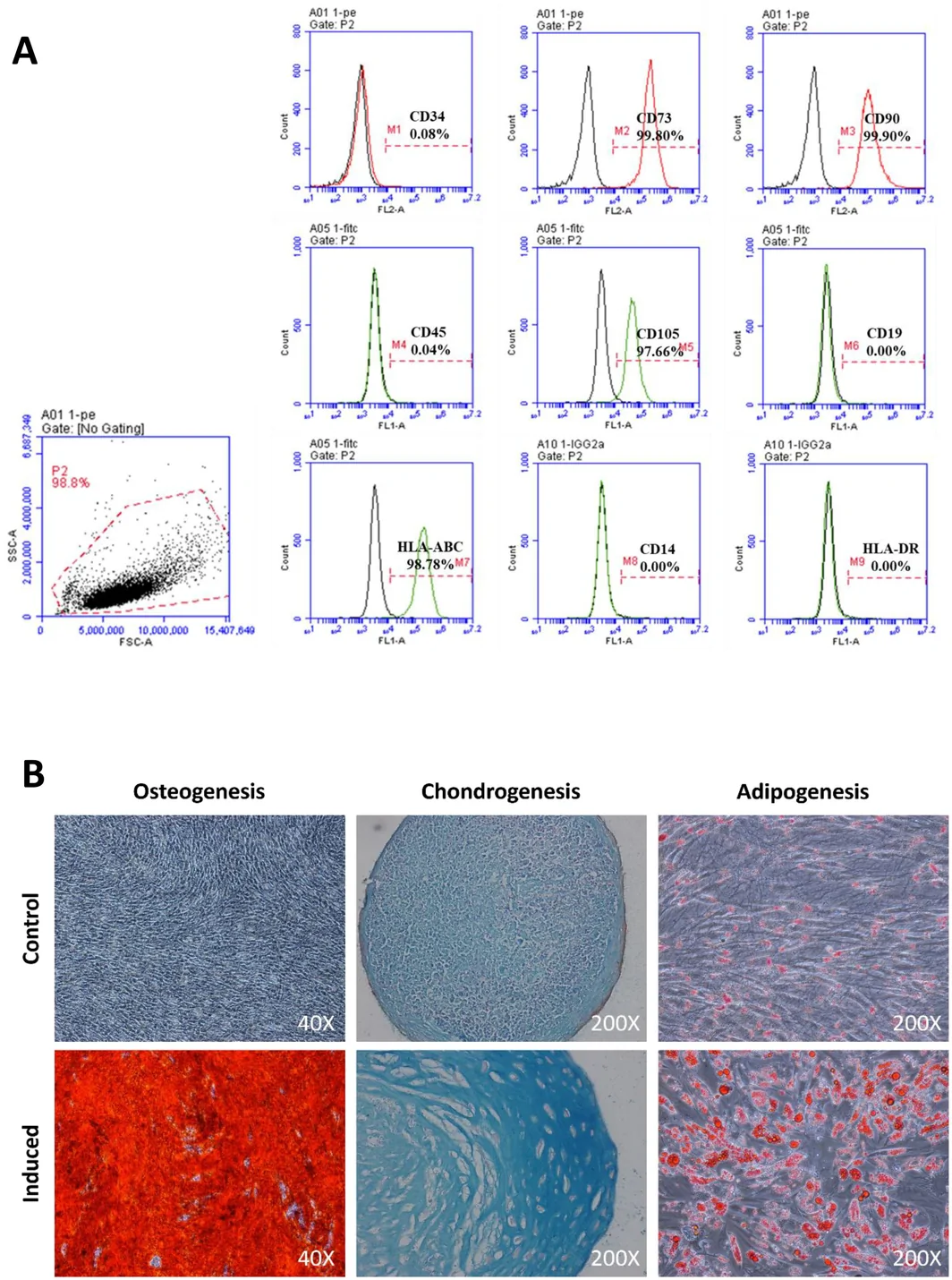
Characterization of human bone marrow‐derived mesenchymal stem cells (hBM‐MSCs). (A) MSCs have been expanded in culture, ex vivo, and phenotypically characterized by flow cytometry. hBM‐MSCs expressed CD73, CD90, MHC class I (HLA‐ABC), and CD105, as determined by positive surface staining. MSCs lack hematopoietic and endothelial markers such as CD14, CD19, CD34, CD45, and MHC class II (HLA‐DR). (B) The capacity to differentiate into multiple mesenchymal lineages, including adipocytes, osteoblasts, and chondrocytes, is used as a functional criterion to define MSCs.

### 3.2 Cell Migration and Immunoregulatory Effects of hBM‐MSCs In Vitro

Built upon the hypothesis that the MSCs possess the capability to induce factors promoting tissue repair and angiogenesis, we evaluated their migration potential using a transwell migration assay with HFFs and HUVECs. As anticipated, the hBM‐MSCs upregulated migration of both HFFs and HUVECs (Figure 2), particularly in the transwell assay with HFFs, where a significant increase in migration compared to the negative control was observed (*p* = 0.002). This result indicates the hBM‐MSCs' ability to contribute to tissue repair in vitro.

**FIGURE 2:**
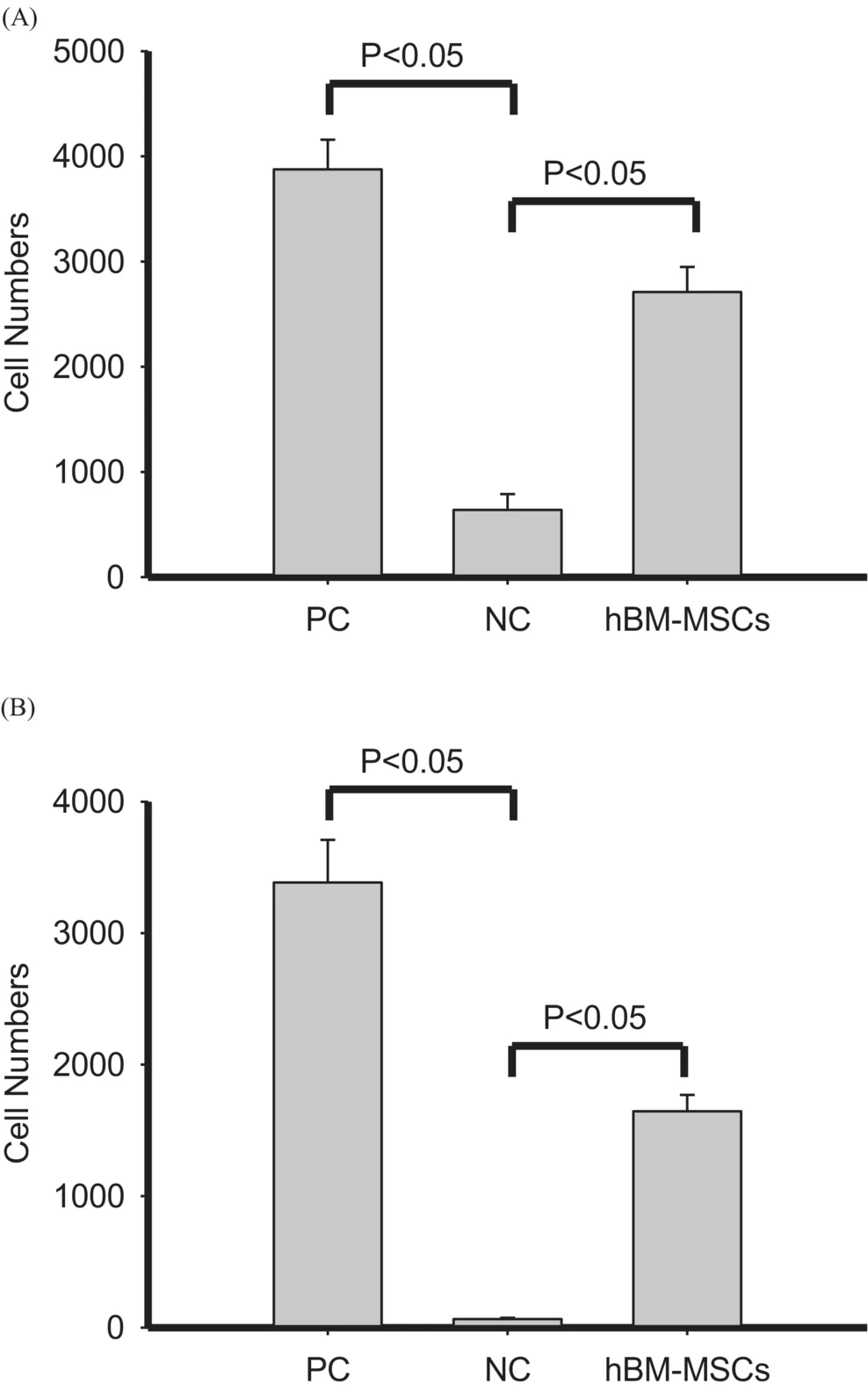
The cell migration rate of human foreskin fibroblasts (HFFs, panel A) and human umbilical vein endothelial cells (HUVECs, panel B) treated with hBM‐MSCs. The data showed the means±SEM (*n* = 3). NC, negative control (medium alone). PC, positive control (medium containing 10% FBS).

### 3.3 hBM‐MSCs Treatment Facilitated Diabetic Wound Healings

To evaluate wound healing, the wounding area was monitored regularly after treatment. The results revealed there were no significant differences in sham with diabetes but no treatment (Group I) and diabetic control with freezing fluids injection alone (Group II) (9.5 ± 1.4 weeks vs. 9.6 ± 1.6 weeks, *p* = 0.934). In contrast, Group III with two sessions of lower dosage of hMSCs can effectively promote wound healing than the experimental control group (sham control) without any treatment (6.3 ± 1.4 weeks vs. 9.5 ± 1.4 weeks, *p* = 0.003) (Figure 3). Group IV revealed a high dosage of hBM‐MSCs showed a statistical increase in wound healing in comparison to controls (5.8 ± 0.8 weeks vs. 9.5 ± 1.4 weeks, *p* = 0.0001). However, there were no significant differences in wound healing time between Groups III and IV. This indicated administration of optimal dosage of hBM‐MSCs has the potential to promote diabetic wound healing.

**FIGURE 3:**
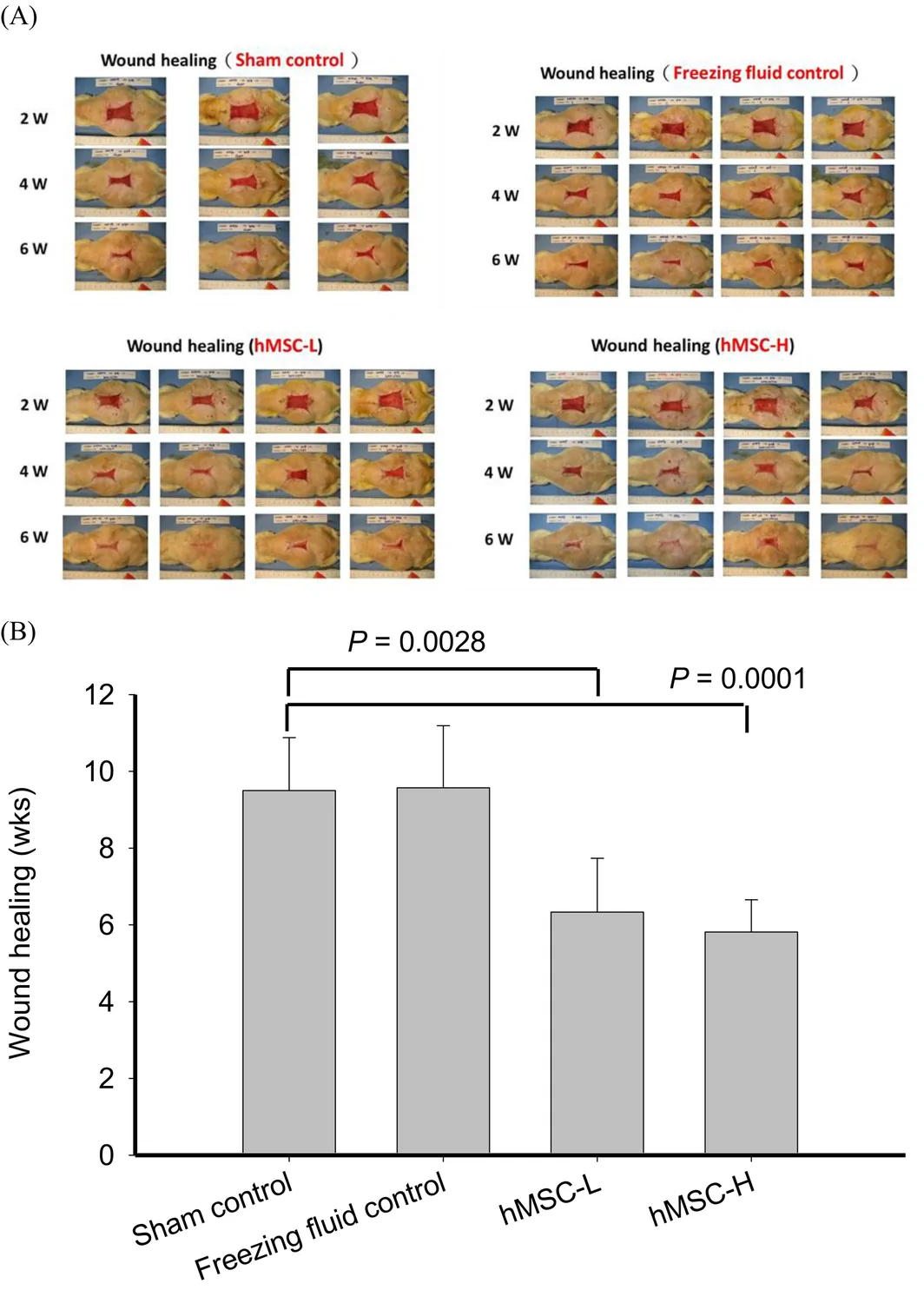
hBM‐MSCs enhanced diabetic wound healing through the recruitment of tissue regeneration in an STZ‐induced diabetes rat. Representative images of the lesion area (initial and from the last day of experimentation) of animals treated with hMSCs (A) and wound closure (%) on 6 weeks post wounding (B). Values in the graph are expressed as means±SD. Sham control (normal); Freezing fluid control (diabetes control without treatment receiving freezing fluid without cells), hMSC‐L (1.4 × 10^6^ MSCs), hMSC‐H (7.7 × 10^6^ MSCs).

### 3.4 hBM‐MSCs Suppressed Inflammatory Response

The specimens harvested from the per wounding area were histologically examined (Figure 4A). CD45 staining revealed that leukocyte infiltration from the dermis to the subcutaneous muscular layers was markedly reduced in Groups III and IV with hBM‐MSCs treated diabetic rat groups on Days 10 and 17 posttreatment compared to that in the diabetic controls (Figure 4B). These results indicate that diabetic rats that received hBM‐MSCs treatments could suppress the early inflammatory responses and facilitate diabetic wound healing.

**FIGURE 4:**
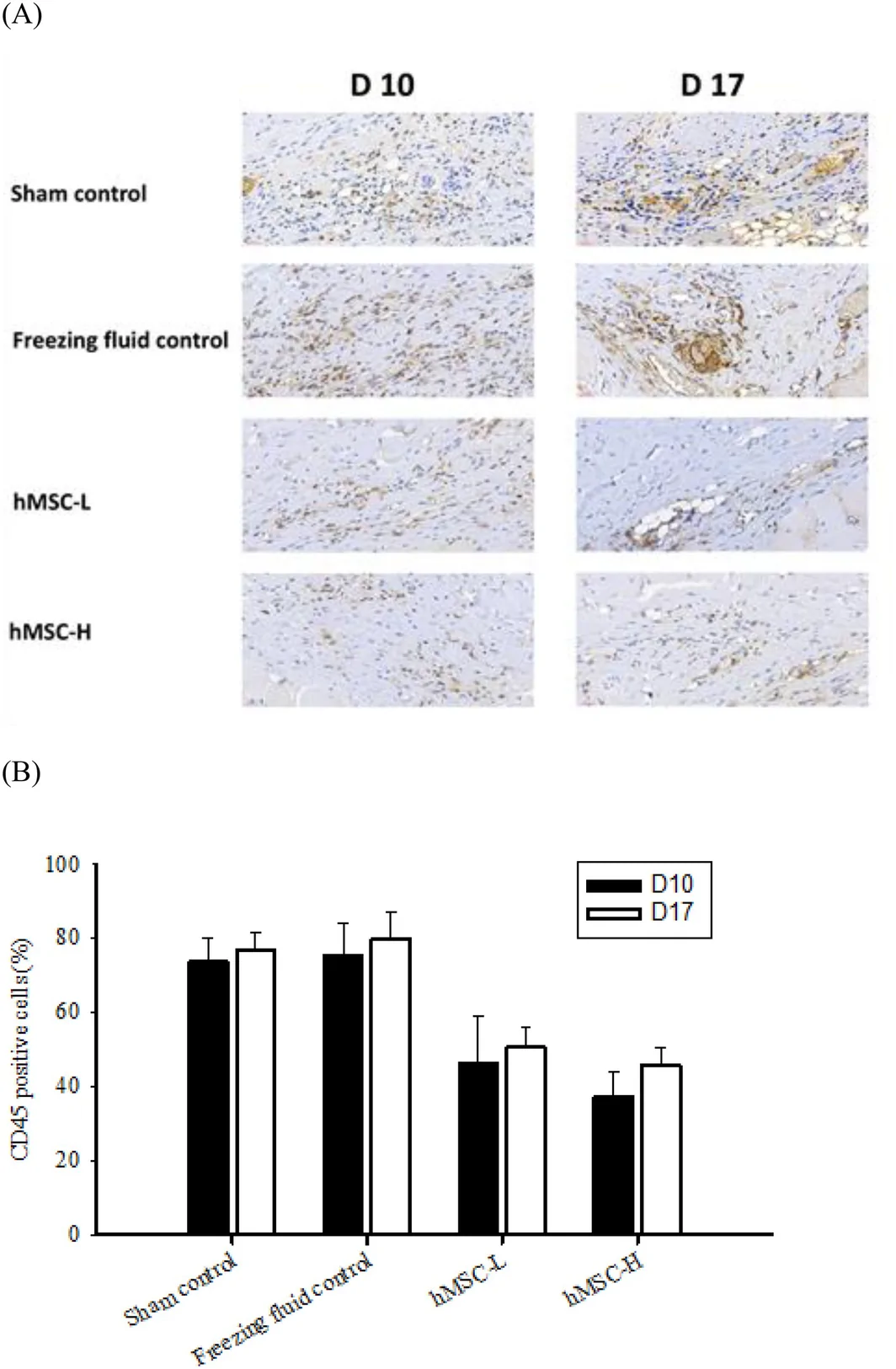
The hBM‐MSCs treatment suppressed the inflammatory responses in wound edges. Histological staining for the CD45 staining to demonstrate the presence of inflammatory cells at the scaffold‐tissue interface (A). Quantification of the CD45 stained layer at the tissue‐scaffold interface (B). Values in the graph are expressed as means±SD. Sham (normal control); Control (diabetes control without treatment receiving freezing fluid without cells), hMSC‐L (1.4 × 10^6^ MSCs), hMSC‐H (7.7 × 10^6^ MSCs).

### 3.5 hBM‐MSCs Treatment Enhanced Cellular Proliferation and Tissue Regeneration

To evaluate cell proliferation and differentiation, Ki‐67 expression was assessed at the wound margins using HRP‐DAB IHC staining. The results revealed a significant increase in Ki‐67 expression in both Groups III and IV with hBM‐MSC‐treated diabetic rats on Day 10 (Table 1 and Figure 5A), particularly in the basal epidermis, dermis, and subcutis, as well as in fibroblasts on Day 17 after treatment. This finding suggests that hBM‐MSCs can enhance cell proliferation at an earlier stage, potentially contributing to accelerated wound healing. Tissue remodeling and collagen processing were examined by the expression of P4H along the wound edge (Figure 5B). The results showed an apparent increase in Groups III and IV with hBM‐MSCs‐treated groups as compared to that in the control group. In contrast, the hBM‐MSC group exhibited a significantly higher expression of EGF at the wound edge compared to the diabetic control group at both Days 10 and 17 posttreatment (Table 1 and Figure 5C). To investigate the angiogenic effects by assessing the VEGF expression in periwound tissues, the results showed the levels of VEGF were significantly increased in the hBM‐MSC‐treated diabetic group posttreatment, particularly in fibroblasts and endothelial cells, as compared to the diabetic control group (Table 1 and Figure 5D). These findings demonstrate that hBM‐MSC treatment in diabetic rats promotes wound healing by enhancing cellular proliferation, angiogenesis, and regeneration.

**TABLE 1:** Summary of Immunohistochemical staining after hMSCs treatment.

	Sham control	Freezing fluid control	hMSC‐L	hMSC‐H	* *p*
Ki‐67					
D10	20.09 ± 4.77	25.76 ± 4.02	42.99 ± 10.43	68.40 ± 6.58	* *p < 0.001*
D17	28.00 ± 11.36	28.56 ± 11.99	41.60 ± 4.92	74.36 ± 5.63	* *p < 0.001*
P4H					
D10	62.68 ± 9.73	64.05 ± 4.77	83.27 ± 7.24	94.38 ± 2.32	* *p < 0.001*
D17	83.59 ± 4.26	87.63 ± 1.73	92.92 ± 1.59	94.95 ± 1.0	* *p < 0.001*
VEGF					
D10	28.85 ± 8.13	29.83 ± 5.19	76.19 ± 7.69	89.72 ± 5.26	* *p < 0.001*
D17	28.71 ± 7.68	39.76 ± 5.79	75.04 ± 6.50	76.08 ± 8.62	* *p < 0.001*
EGF					
D10	19.62 ± 5.53	17.28 ± 7.13	42.55 ± 14.07	56.41 ± 7.38	* *p < 0.001*
D17	24.71 ± 7.02	28.44 ± 5.69	62.25 ± 10.35	67.41 ± 11.71	* *p < 0.001*

Abbreviations: D10, 10 days postoperatively; D17, 17 days postoperatively; EGF, epithelial growth factor; hMSC‐H, high dose, 1 × 10^7^ MSCs; hMSC‐L, human bone marrow‐derived MSC (low dose, 2 × 10^6^ MSCs); Ki‐67, proliferation marker; P4H, prolyl 4‐hydroxylase; VEGF, vascular endothelial growth factor.

* One‐way analysis of variance was performed to determine the significant differences in groups with a lognormal distribution. Post hoc comparison was performed using the Holm–Šidák method, and values of *p* < 0.05 were considered statistically significant.

**FIGURE 5:**
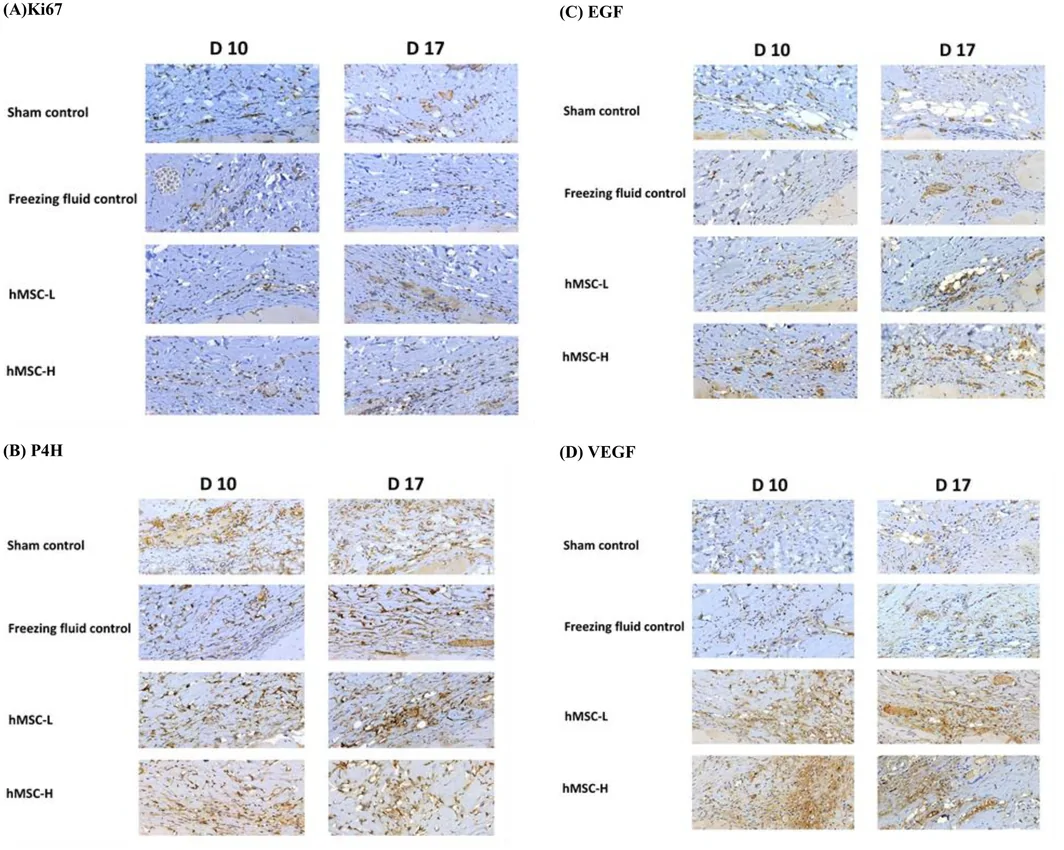
hBM‐MSCs treatment upregulated cellular proliferation and regeneration. (A) Immunohistochemical staining indicated that the cellular proliferation marker, Ki‐67, expression levels in the wound edge were visibly increased in the hBM‐MSCs group on Day 10 compared to that of the diabetic controls (original magnification, ×100). (B) Immunohistochemical staining indicated that prolyl 4‐hydroxylase (P4H) expression was significantly increased in the hBM‐MSCs group on Day 10 compared to that of the controls. (C) Immunohistochemical staining indicated that VEGF expression was significantly increased in the hBM‐MSCs group on Day 10 compared to that of the controls. (D) Immunohistochemical staining indicated that EGF expression was significantly increased in the hBM‐MSCs group on Day 10 compared to that of the controls. Sham (normal control); Control (diabetes control without treatment receiving freezing fluid without cells), hMSC‐L (1.4 × 10^6^ MSCs), hMSC‐H (7.7 × 10^6^ MSCs).

## 4 Discussion

Despite the development of numerous therapies for chronic wound healing, some results are still controversial and challenging due to various reasons. Over the past several decades, MSCs have been investigated for their impact on wound healing and seem to emerge as a promising approach. Studies showed the effects of MSCs rely on their immunomodulatory and proangiogenic characteristics [19, 20]. Moreover, MSCs have been shown to mediate neovascularization through angiogenesis [7, 21]. Our prior animal research, utilizing a rodent dorsal wound model, showcased the enhanced healing and closure of diabetic wounds following the topical administration of allogenic rodent normal BM‐MSCs [9] and rodent ADSCs [8]. Bone marrow serves as a rich source of diverse stem cells, including hematopoietic stem cells (HSCs) and MSCs, each playing a unique role in blood cell formation and tissue regeneration. The advantageous attributes of BM‐MSCs lie in their innate ability to modulate the immune response, stimulate angiogenesis, and promote tissue repair, making them particularly well‐suited for wound‐healing applications [22]. These positive effects were characterized by heightened epithelialization and granulation tissue production, suggesting a combination of increased fibroblast proliferation, augmented growth factor production, and enhanced cellular recruitment contributed to the observed improvements. In this study, the application of allogenic hBM‐MSCs demonstrated a positive effect, further enhancing diabetic wound healing.

The clinical application of human autologous mesenchymal stem cells (auto‐MSCs) faces several potential limitations. Firstly, obtaining a sufficient number of autologous MSCs from certain patients can be challenging, such as from lean individuals for ADSCs or from myelofibrosis patients for BM‐MSCs. Secondly, MSCs isolated from elderly donors exhibit reduced biological activity, including differentiation and regenerative potential, potentially leading to suboptimal therapeutic outcomes. Thirdly, certain systemic diseases, including diabetes, rheumatoid arthritis, and systemic lupus erythematosus, can alter the intrinsic properties of MSCs, impairing their protective functions [23, 24, 25, 26]. Consequently, obtaining a sufficient quantity of high‐quality autologous MSCs from patients with these conditions can be difficult, posing challenges for MSC transplantation. The procurement of allogeneic mesenchymal stem cells (allo‐MSCs) from young, healthy donors offers a viable solution to address these limitations. Unlike auto‐MSCs, which require time‐consuming extraction procedures, allo‐MSCs are readily available and can be promptly administered for acute conditions such as stroke or myocardial infarction [26]. Additionally, commercialized allo‐MSC production can ensure quality control and reduce the overall cost of cell therapy. As a result, allo‐MSCs present a promising alternative to auto‐MSCs, offering advantages in terms of timeliness, cost‐effectiveness, and quality assurance.

To validate the safety and efficacy of allo‐MSCs in wound healing, a diabetic wounding animal model was employed. Our results showed diabetic rats treated with hBM‐MSCs postwounding, no matter the lower dosage or higher dosage, both exhibited significant enhancement of wound healing, as compared to the untreated control group. Stem cell therapy activates various key cellular processes for damaged tissue regeneration. A previous investigation highlighted the pivotal role of leukocyte‐mediated inflammation in influencing the wound‐healing process [9]. Our present study, through histological analysis of periwound tissues, revealed that inflammatory cell (CD45^+^ cells) infiltration was attenuated in the hBM‐MSCs‐treated group compared to controls. These findings demonstrated that hBM‐MSCs enhanced wound healing by suppressing topical proinflammatory cellular responses in the wound healing process. Immunohistochemical analysis revealed enhancement of cellular proliferation and regeneration in the wound margin area of the hBM‐MSCs‐treated group. Increased expression of Ki‐67 and P4H, particularly in fibroblasts and basal epidermal layers, was observed, respectively. This indicated hBM‐MSCs‐treated could significantly enhance cell proliferation and tissue regeneration. Additionally, angiogenesis plays a key role in wound healing, being especially important in chronic ulcers, by restoring blood perfusion and delivering nutrients to injured sites. Our results showed EGF expression significantly increased in the wound margin area, and VEGF expression increased in the periwound edge, particularly in endothelial cells and fibroblasts after hBM‐MSCs application. These findings suggested that BM‐MSCs enhanced wound epithelialization and neovascularization through increased expression of growth factors such as EGF and VEGF, promoting epithelialization in the periwound zone.

Although our results showed superior cellular hormone indicators in the higher‐dose hBM‐MSC group as compared to the lower‐dose hBM‐MSC group, our results did not show a significant difference to facilitate the wound healing in the higher‐dose hBM‐MSC group as compared to the lower‐dose hBM‐MSC group. Further optimal dosage of hBM‐MSCs for human clinical trials and elucidate the biomechanisms are still needed.

In conclusion, subcutaneous injections of hBM‐MSCs demonstrated acceleration of diabetic healing compared to the untreated control group, without any adverse reactions. IHC staining indicated that hBM‐MSCs effectively inhibited wound inflammation and promoted the processes of cellular proliferation and differentiation, enhancing angiogenesis and tissue remodeling. Overall, hBM‐MSCs exhibit promising potential for promoting diabetic wound healing with good safety profiles. These results provide an effective alternative therapeutic strategy for patients with chronic wounds who cannot benefit from autologous cell therapies.

## Funding

This work was partly supported by the National Science and Technology Council, NSTC, Taiwan (114‐2314‐B‐037‐080) and the Kaohsiung Medical University Hospital, Taiwan (KMUH111‐1M25, KMTTH‐113‐R005). The authors thank the Center for Laboratory Animals in Kaohsiung Medical University for the animal care.

## Conflicts of Interest

The authors declare no conflicts of interest.

## Data Availability

The data that support the findings of this study are available from the corresponding author upon reasonable request.
